# Investigation of GIM-TEC disturbances before M ≥ 6.0 inland earthquakes during 2003–2017

**DOI:** 10.1038/s41598-020-74995-w

**Published:** 2020-10-22

**Authors:** Fuying Zhu, Yingchun Jiang

**Affiliations:** 1 Key Laboratory of Earthquake Geodesy, Institute of Seismology, China Earthquake Administration, Wuhan, 430071 China; 2 Wuhan Base of Institute of Crustal Dynamics, China Earthquake Administration, Wuhan, 430071 China; 3 College of Logistics Management, Hubei Urban Construction Vocational and Technological College, Wuhan, 430205 China

**Keywords:** Geophysics, Natural hazards

## Abstract

With the rapid development of the Global Navigation Satellite System (GNSS) and its wide applications to atmospheric science research, the global ionosphere map (GIM) total electron content (TEC) data are extensively used as a potential tool to detect ionospheric disturbances related to seismic activity and they are frequently used to statistically study the relation between the ionosphere and earthquakes (EQs). Indeed, due to the distribution of ground based GPS receivers is very sparse or absent in large areas of ocean, the GIM-TEC data over oceans are results of interpolation between stations and extrapolation in both space and time, and therefore, they are not suitable for studying the marine EQs. In this paper, based on the GIM-TEC data, a statistical investigation of ionospheric TEC variations of 15 days before and after the 276 M ≥ 6.0 inland EQs is undertaken. After eliminating the interference of geomagnetic activities, the spatial and temporal distributions of the ionospheric TEC disturbances before and after the EQs are investigated and compared. There are no particularly distinct features in the time distribution of the ionospheric TEC disturbances before the inland EQs. However, there are some differences in the spatial distribution, and the biggest difference is precisely in the epicenter area. On the other hand, the occurrence rates of ionospheric TEC disturbances within 5 days before the EQs are overall higher than those after EQs, in addition both of them slightly increase with the earthquake magnitude. These results suggest that the anomalous variations of the GIM-TEC before the EQs might be related to the seismic activities.

## Introduction

The Global Navigation Satellite System (GNSS) has been used as a valuable tool to monitor and estimate ionospheric total electron contents (TECs)^1–5^. The magnitude of the TEC is variable and depends on several factors such as geomagnetic storm, season, local time, the solar activity and seismic activity^6–10^. A large number of excellent papers published in journals have shown that the ionospheric disturbances exist prior to strong EQs^8,9,11–16^, including electromagnetic anomalies, plasma density, temperature changes, etc. However, due to the limitation of observation technology, the ionospheric TEC derived from ground-based GNSS receivers has attracted much attention of scientists^14,15,17^. Nowadays dense and continuous observation has been achieved globally, the International GNSS Service (IGS) has ensured the availability of open access, high-quality GNSS TEC products, IGS Global Ionosphere Map (GIM) TEC data are a superset of data to monitor and study the phenomenon of seismic ionospheric disturbances. Especially in recent years, in order to search for a possible relation between the ionosphere and the seismic activity, based on the GIM-TEC data, more and more statistical analyses with a lot of events have been performed for EQs in some regional areas^16–25^ or global EQs^26–31^. However, the pre-earthquake ionospheric disturbances are still challenging and controversial^21,31–33^. In the same time, validated physical generation mechanisms of possible ionospheric disturbance are not yet identified. On the other hand, most of the previous statistical analyses targeted all kinds of EQs, including inland EQs and EQs in the ocean. However, the GIM-TEC values over oceans are result of interpolation between stations and extrapolation in both space and time, and the formal error of low latitude values reach a maximum of about 10 TECu^2^, where 1 TECu = 10^16^ el/m^2^. Therefore, they are not suitable to study the marine EQs, that is to say, the foregoing statistical studies about the ionospheric TEC disturbances before the EQs (mainly marine EQs) are strictly unreliable.

In this paper, in order to test a possible statistical significance between the ionospheric TEC disturbances and the seismic activities, particularly for the global inland EQs (M ≥ 6.0), we carried out a statistical study by investigating the spatial–temporal distribution of the GIM-TEC disturbances.

### Data sets

**Figure 1:**
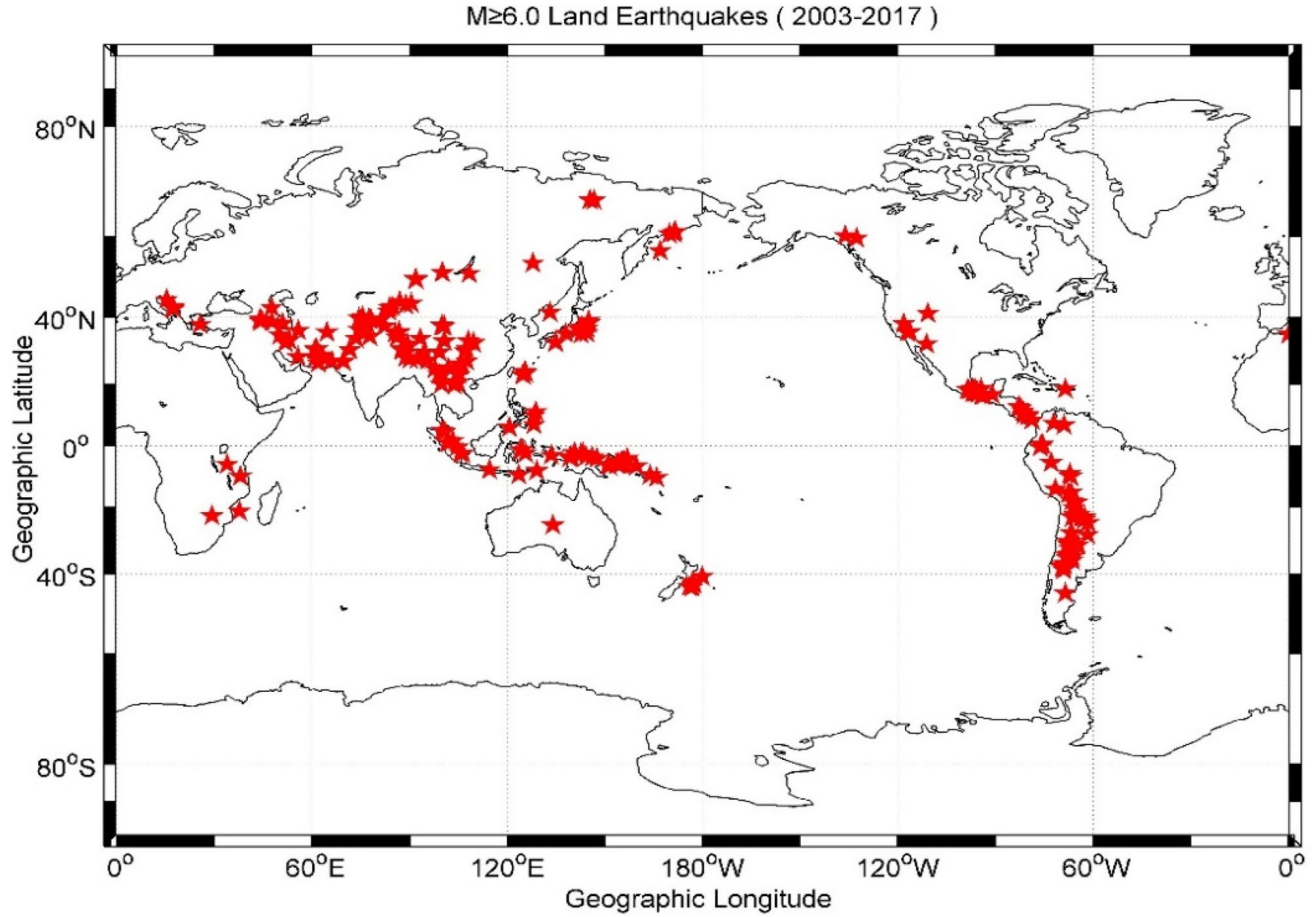
Map of 276 M ≥ 6.0 inland earthquakes (red pentacles) data set during 2003–2017. The figure was generated in MATLAB version R2014b (8.4.0.150421) (https://www.mathworks.com/products).

In this study, we used the IGS GIM-TEC, which are constructed with more than 2000 worldwide ground based GNSS receivers and have a 2 h temporal resolution and a spatial resolution of 5.0° × 2.5° in longitude and latitude^4^, and therefore each map consists 5183 (= 73 × 71) grid points. Indeed, in marine regions there are few GNSS receivers, and as a consequence, most part of the provided information by the TEC global map is based on interpolation, it is not suitable to study the marine EQs. In order to hunting the correlation between the ionospheric TEC disturbances and the seismic activities, we only select the M ≥ 6.0 shallow depths (≤ 60 km) inland EQs around the world from 2003 to 2017 in this study, the EQs data are retrieved from the EQ catalogue of United States Geological Survey (USGS) through the following address (https://earthquake.usgs.gov/earthquakes). The epicenters of these inland EQs are illustrated in Fig. 1, the red pentacles denote the M ≥ 6.0 inland EQs.

## Methods

To identify possible ionospheric TEC disturbance, we still select the running window statistical analysis which is applied in detection of TEC disturbance in many studies^17,28,29^. Several steps are to be followed to establish the reliable methodology. The first step is to extract the GIM grid point nearest to an epicenter as the earthquake associated point and calculated its TEC variation. The second step is make further effort to take out the long-term trend of the TEC time-series by filtering. The third step is our final goal, that is, to determine the TEC disturbance. After the second step, we computed, as usual, the mean ($\upmu = \stackrel{-}{\mathrm{x}}$) and the standard deviation (σ) at each time point based on the previous 15-day TECs, and then we set $\mathrm{UB}=\upmu +2\upsigma$ and $\mathrm{LB}=\upmu -2\upsigma$, as the upper bound and the lower bound, respectively. If an observed TEC value fell outside the associated LB or UB, we then declare that a negative or positive disturbance being detected^17,28^. It is well known that solar and geomagnetic activity can affect the ionosphere. So the fourth necessary step is to exclude the interference of the solar and geomagnetic activity. That is, we analyzed the Kp and Dst indices of the same period^27,28^. If Kp >  = 4 or *Dst* < − 40 nT (or the decrease of Dst within a day is larger than 40 nT) occurred, all the TEC disturbances on this day and the following 3 days will be abandoned.

In order to clearly demonstrate the spatial–temporal distribution of the ionospheric TEC disturbances, we studied in detail the occurrence rates of the TEC disturbances 15 days before and after all the 276 inland EQs. Here, the occurrence rate is defined as the ratio of the number of EQs before which at least one TEC disturbance exists to the total number of all the EQs. That is if the number of EQs before which there are TEC disturbances occurred in a certain period of time (e.g., 1 day) is N, then the occurrence rate of the ionospheric TEC disturbance at this time interval can be calculated as N/276 × 100%.

## Results

### Temporal analysis

**Figure 2:**
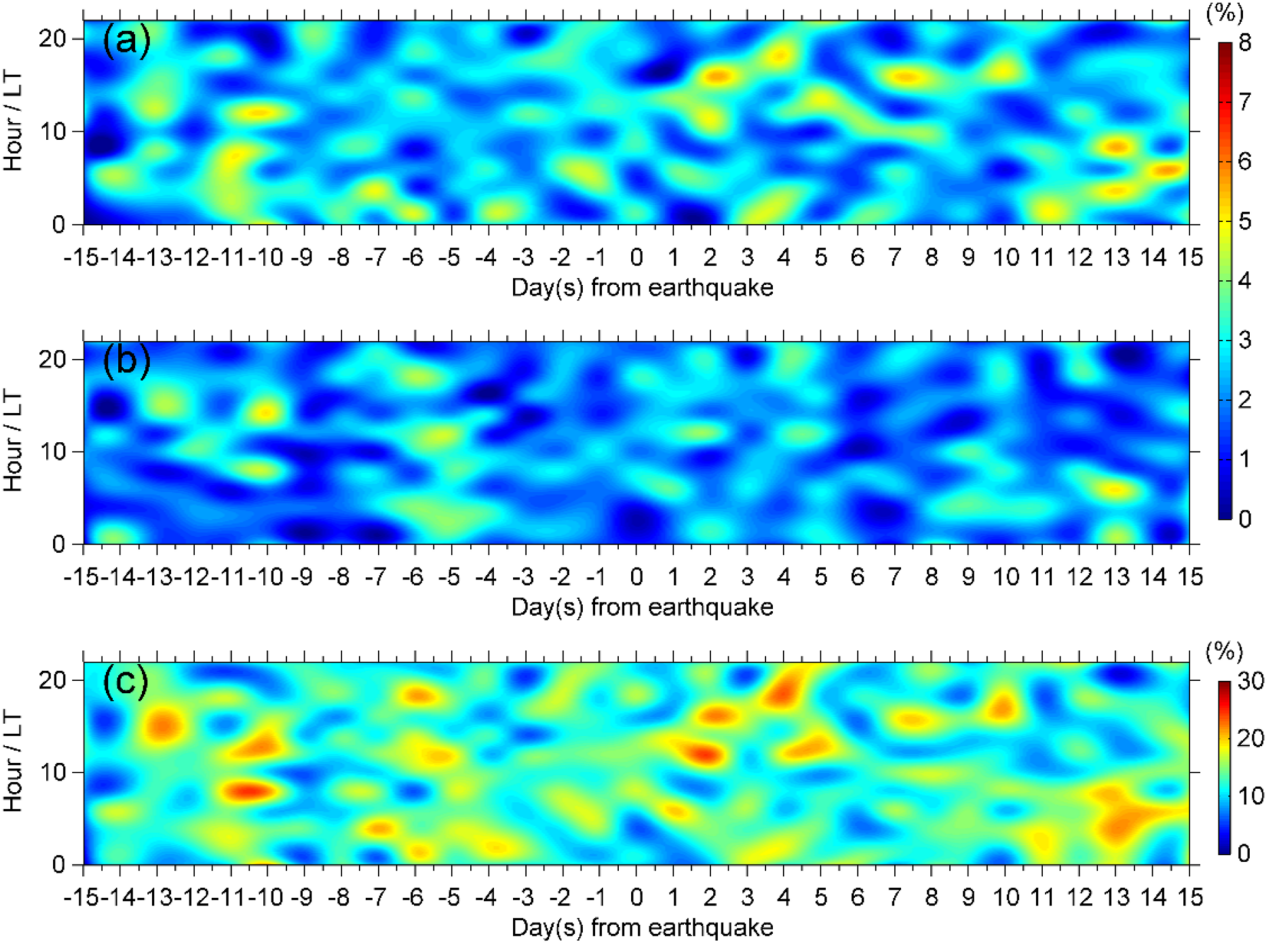
Temporal distribution of ionospheric TEC disturbances 15 days before and after the inland EQs during 2003–2017. (**a**,**b**) show the event percentage of TEC positive disturbance and negative disturbance at every moment, respectively, (**c**) shows the event percentage of all TEC disturbances.

According to the method described above, to establish the correlation between the GIM-TEC disturbances and the seismic activities, we statistically investigated the occurrence rates of the every ionospheric TEC value 15 days before and after all the 276 inland EQs. Figure 2 shows the temporal distribution of occurrence rates of all the EQs. From the top to the bottom, the panels are related to the occurrence rates of the ionospheric TEC positive disturbance, the occurrence rates of the ionospheric TEC negative disturbance and the occurrence rates of the ionospheric TEC disturbance (including positive disturbance or negative disturbance). The horizontal axis denotes days before EQ, the vertical axis denotes local time, and color of each grid represents the occurrence rate of TEC disturbance. From Fig. 2, one can see that the occurrence rates of TEC positive disturbance, especially the occurrence rates of negative disturbance are generally very low (less than 6%), and the GIM-TEC disturbances appear without a certain law 15 days before and after the inland EQs. That is to say, the chances of detecting the ionospheric disturbance in TEC seem not to be a function of time. It is clear that, the max occurrence rate of TEC disturbance is 24% which occurred at 1400LT on the 2th day after earthquake. Comparing to pre-seismic effects, post-seismic effect is more apparent correspond to the former conclusions^28,29,34^. In short, there are no obvious features in time distribution of TEC disturbances before the inland EQs.

**Figure 3:**
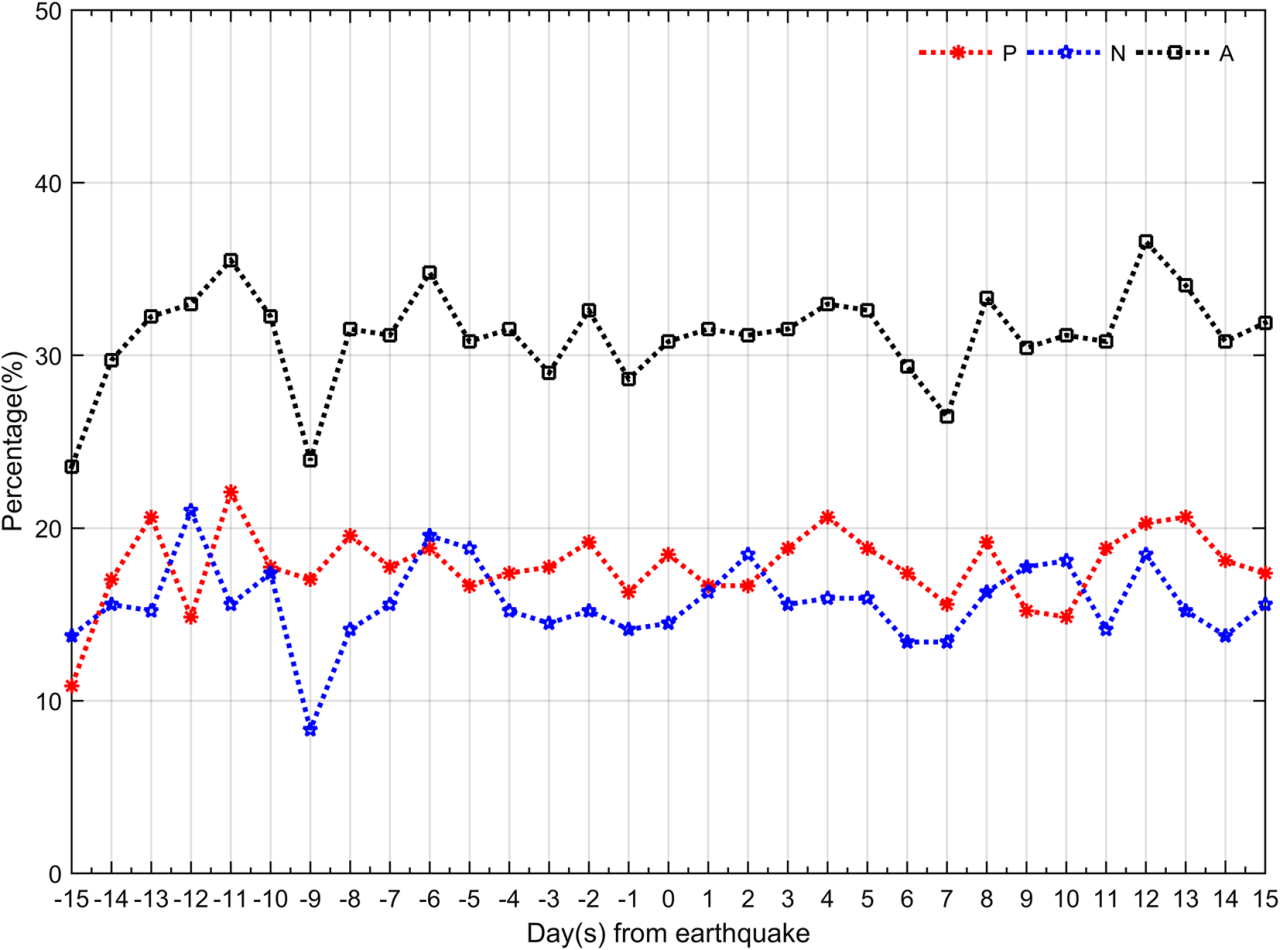
Temporal distribution of event percentages of the TEC disturbances everyday.

To compare the ionosphere disturbance behaviors before and after inland EQs, the event percentages of TEC disturbance detected at every moment are stacked every day, respectively. Figure 3 reveals the cumulative event percentages of the occurrence rates of TEC disturbances 15 days before and after the EQs, The red grains denote the ionospheric TEC positive disturbance, the blue stars denote TEC negative disturbance and the black rectangles denote all TEC disturbance. As is seen in the Fig. 3, the time distribution of the TEC positive and negative disturbances every day before and after EQs is similar to that in Fig. 2, since the percentage of day is stacked with the percentage of hour, it is clear that these occurrence rates of event percentages are overall higher than those in Fig. 2. and the occurrence rates of ionosphere disturbance behavior before and after the EQs is also not high. The average event percentage of the TEC positive disturbance and negative disturbance is 17.78% and 15.70%, respectively. From Fig. 3, one can also see that the difference of the occurrence rate of the between before and after the EQs is very small. In general, similar to previous conclusions^24,28,29,31^, we find no evidence of significant changes in the GIM-TEC disturbances prior to the inland EQs in this statistical analysis. This is because that it is difficult to clearly distinguish the pre-seismic ionospheric TEC variation, which can be supposed as possible precursors. Of course, this result does not completely deny the possibility of ionospheric precursors.

### Spatial analysis

**Figure 4:**
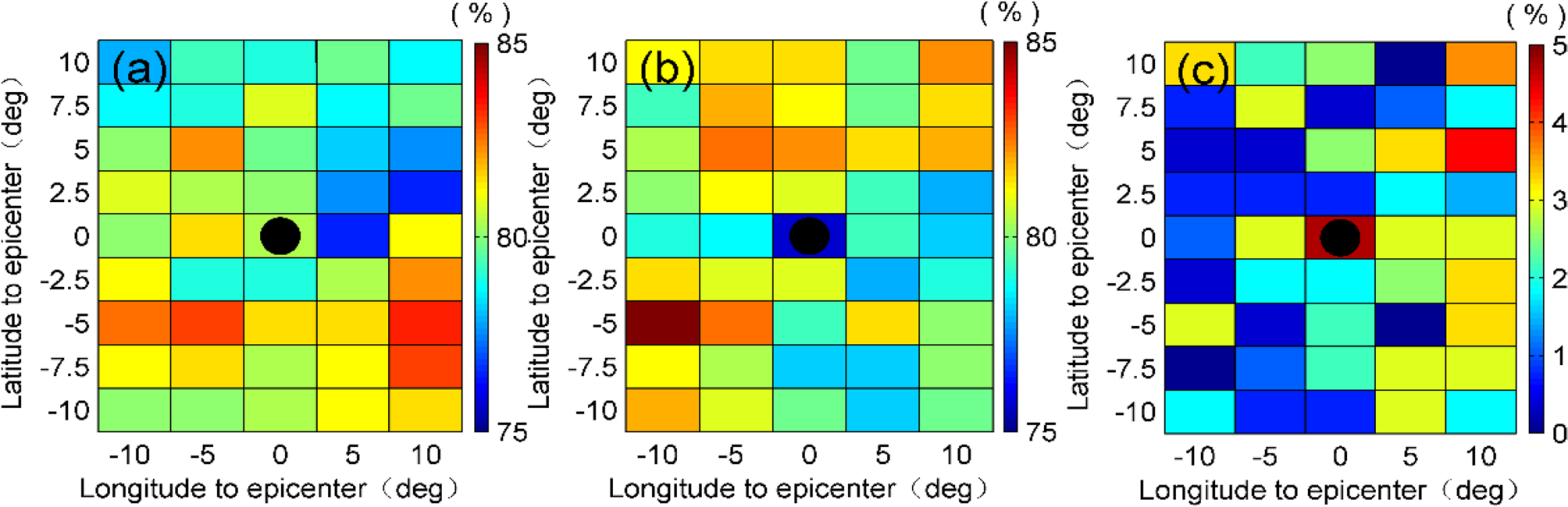
Spatial distribution of the occurrence rates of the ionospheric TEC disturbances. (**a**,**b**) show the occurrence rates of the ionospheric TEC disturbances from 1 to 5 days before and after the EQs, respectively. (**c**) shows the difference between (**a**) and (**b**). The x-axis is the geographic longitude and the y-axis is the geographic latitude for all of the images. Color of each grid represents the occurrence rate of TEC anomalies, and the black circles denote epicenters.

In order to search for a possible relation between the ionospheric disturbances and the seismic activities, in this section, we are about to further investigate the spatial characteristics of the ionospheric TEC disturbances. Pulinets and Boyarchuk^13^ have suggested that the ionospheric concentration gradually started to diminish 5 days before the seismic shock, and a great many studies have shown that the seismo-ionospheric disturbances most likely appear 1–5 days prior to earthquakes^15,18,19,23,26,29,30^. Therefore, in the present analysis it is considered that pre- and post- 5 days data are influenced by seismogenic effects. We firstly detect the TEC disturbances over the region of ± 10° nearest an epicenter, then the occurrence rates of ionosphere disturbances from 1 to 5 days before or after the earthquakes are stacked for all the selected EQs. Figure 4 shows the spatial distribution of occurrence rates of the TEC disturbances.

As you can see from the Fig. 4a,b, the occurrence rates of the pre-seismic and post-seismic TEC disturbances within 1–5 days are not particularly significant, the occurrence rates of TEC positive disturbances mainly appear not over the epicenters but on the south sides of epicenters. To be specific, the maximum detection probability of TEC disturbance before earthquake is mainly on the southwestern and southeastern direction to the epicenters, which locate in 5° from the epicenters. However, for the post-seismic ionospheric TEC disturbances, the larger occurrence rates mainly appear on the southwest sides of epicenters, which is basically consistent with Liu and Wan^24^. One of possible reasons is due to the fact that the epicenters of EQs they studied also located on land. Moreover, the results also show that the maximum difference of the occurrence rates of the pre-seismic and post-seismic TEC disturbances appears over the epicenter. The reason may be due to the fact that there is indeed a potential connection between EQs and these ionospheric disturbances.

**Figure 5:**
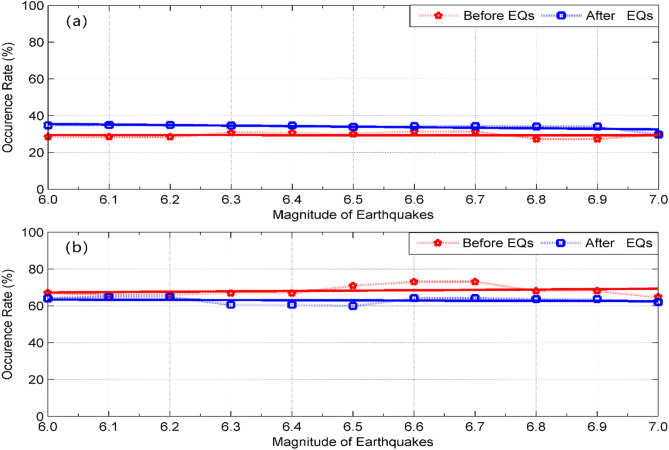
Occurrence rates of the TEC disturbances appear within 1 day (**a**) and 5 days (**b**) before and after the earthquakes with different magnitudes. The red and blue colors denote TEC disturbances before EQs and after EQs, respectively. The solid lines denote the fitting curves.

Finally, in order to investigate whether the ionospheric TEC disturbances are related with the earthquakes or not. We also made statistical calculation and comparison . Figure 5a,b show the occurrence rates of the TEC disturbances appear within 1 day and 5 days before and after the EQs with different magnitudes, respectively. From Fig. 5a, one can see that the occurrence rates of the TEC disturbances appear within 1 day after EQs are all higher than those before EQs, in other words, comparing to the pre-seismic effects, the post-seismic effects are more apparent. However, it is apparent in Fig. 5b that the occurrence rates of the TEC disturbances within 1–5 days before EQs are larger than those after EQs, the pre-earthquake ionospheric TEC disturbances occur more frequently (the occurrences rate is about 70% ) with the earthquake magnitude than those after EQs. The fitted curves presented in Fig. 5b indicate that the occurrence rate of the TEC disturbances increase with the increase of earthquake magnitude, it may be a linear function of the earthquake magnitude with positive slope. It’s clear that a greater earthquake shall yield more energy. Therefore, the larger the earthquake, the better chance observing the seismo-ionospheric disturbance which agrees well with the previous results reached by Liu et al.^12,26^. In other words, this finding demonstrates that the occurrence rate of the ionospheric TEC disturbances is proportional to the earthquake magnitude. We thus conclude that with the increase of earthquake magnitude, the slight increase of the occurrence rates of the TEC disturbances before EQs is likely related to the forthcoming seismic activity.

## Discussion

We tend to look for the possible impact of land-based seismic activities on the ionosphere through large sample statistical analysis. As we all know that the coupling process of earthquake ionosphere is a complex physical phenomenon^13,35^. Up to now, the actual generating mechanism is still not understood comprehensively. The variations of ionospheric TEC parameters are not only due to EQs, and there are numerous possibilities of ionospheric disturbances which could come from other sources (e.g., solar activity, acoustic gravity waves, travelling ionospheric disturbances, plasma dynamics). Accordingly, how to identify effectively the seismic-ionosphere is the most challenging. Then the GIM-TEC maps are interpolated in both space and time^2^. If the GNSS receiver is very sparse or absent near an earthquake, the seismo-ionospheric disturbances will not be objectively reflected. Meanwhile, most of the EQs are concentrated around the low latitude, some are in equatorial regions. Simple statistical processing can mix these effects, as a result, we may obtain zero as output. In addition, the number of EQs with individual magnitude is still small, a larger sample catalogue is needed to obtain more meaningful statistical features. This paper mainly gives some preliminary results which may shed light on the future study for the investigation of the ionospheric precursors of EQs. In order to study the interaction between seismicity and ionosphere, it is necessary to make more detailed statistical analysis of more earthquake cases in the future.

## Conclusion

In the present paper, in order to seek a possible statistical significance between the TEC disturbances and the seismic activities, we statistically investigated the space–time distributing of pre-seismic and post-seismic changes in GIM-TEC for 276 global M ≥ 6.0 inland EQs during 2003–2017. There is no significant difference in the time distribution of positive and negative ionospheric disturbances before and after EQs. However, there are some weak differences in spatial distribution, and the biggest difference lies in the epicenter area. Moreover, our results show that the occurrence rates of ionospheric TEC disturbances 1–5 days before EQs are higher than those after EQs, and both of them slightly increase with the magnitude. Again, these conclusions reflect to some extent that there is still a specific unknown correlation between the EQs and the ionospheric disturbances.

## Data Availability

The dataset will be available from the corresponding upon request.
